# Impact of multiple injuries on functional and neurological outcomes of patients with spinal cord injury

**DOI:** 10.1186/1757-7241-21-42

**Published:** 2013-05-30

**Authors:** Giorgio Scivoletto, Sara Farchi, Letizia Laurenza, Federica Tamburella, Marco Molinari

**Affiliations:** 1 Spinal Cord Unit, IRCCS S. Lucia Foundation, Rome, Italy; 2 Clinical and Research Movement Analysis (CaRMA) Lab, IRCCS S. Lucia Foundation, Rome, Italy; 3 Public Health Agency of Lazio Region, Rome, Italy

**Keywords:** Spinal cord injury, Multiple injuries, Outcomes

## Abstract

**Background:**

The effects of multiple injuries on the neurological and functional outcomes of patients with traumatic spinal cord injury (SCI) are debated—some groups have shown that subjects with multiple injuries have the same neurological and functional outcomes of those without them, whereas others have found that SCI patients with associated traumatic brain injury have worse functional status at admission and discharge and longer rehabilitation stays than patients without brain injury. Thus, the aim of this study was to compare the outcomes of SCI subjects with or without multiple injuries.

**Methods:**

A total of 245 patients with a traumatic SCI during the first rehabilitation stay after the development of the lesion (202 males and 43 females; age 39.8 ± 17 years; lesion to admission time 51.1 ± 58 days) were examined on a referral basis. Patients were assessed using the following measures: American Spinal Injury Association standards, Barthel Index, Rivermead Mobility Index, and Walking Index for Spinal Cord Injury. The statistical analysis comprised Poisson regression models with relative risks and 95% confidence intervals, adjusted for the following confounders: age, sex, lesion level, and ASIA impairment scale (AIS) grade. Student’s *T* test was used to compare the outcomes of patients divided by AIS impairment and lesion level.

**Results:**

SCI patients with and without multiple injuries differed significantly with regard to the level and completeness of the lesion. Overall, patients with multiple injuries had worse functional status at admission and discharge than monotraumatic subjects. However, when adjusted for neurological features, the populations had comparable functional and neurological status at admission and discharge and similar rates of complications and discharge destinations. The separate analysis per each level of lesion/AIS grade showed that in some groups, patients with multiple injuries had a significant longer length of stay or worse functional status at rehabilitation admission (but not at discharge) than their monotraumatic counterparts.

**Conclusions:**

Multiple injuries do not affect the neurological or rehabilitative prognosis of spinal cord injuries. At discharge, patients with spinal cord injuries with and without multiple injuries achieved similar results with regard to neurological and functional improvement. Some groups of patients with multiple injuries had a longer length of stay.

## Introduction

The prediction of neurological and functional outcomes after development of a spinal cord lesion (SCL) is essential to answer patients’ questions regarding their functional potential and to understand the breadth of resources that is required during inpatient rehabilitation and after discharge. Further, precise knowledge of the course of the natural recovery of SCL and the factors that affect it is necessary to evaluate the efficacy of new pharmacological and rehabilitative strategies [1,2].

Several studies have examined the effects of various factors on neurological and functional recovery after SCI, such as age [3], neurological status at admission [4], and rehabilitation timing [5]. Although a high percentage of patients with a traumatic SCI present with multiple injuries [6,7], few studies have investigated the effects of multiple injuries on the outcome of SCIs, most of which have merely addressed the issue of surgery and surgical efficacy in these patients [8,9]. Only 3 studies [10-12] have reported the outcomes of SCI patients with and without multiple injuries in a small cohort of paraplegic patients with complete lesion.

The aim of this study was to examine the functional and neurological status of SCI patients with and without multiple injuries at admission and discharge and the factors that are associated with functional status in patients with spinal cord lesions. We hypothesized that multiple injuries negatively affect the functional status of such patients (particularly at admission) and the length of rehabilitation stay.

## Patients and methods

We retrospectively examined the charts of 245 patients with traumatic spinal cord injury who were admitted to our spinal unit between 1996 and 2011 for their first rehabilitation treatment after development of the lesion. Patients were admitted on a referral base. The inclusion criteria for admission to our unit were age over 12 years, being clinically stable, and having finished the surgical workout. The few cases that experienced progression of the lesion were excluded. Whenever a patient was discharged or had been transferred for more than 3 weeks, the readmission was considered a second admission, and the patient was excluded. The study was approved by the Institutional Review Board of the IRCCS S. Lucia Foundation. The research was carried out in compliance with the Helsinki Declaration.

The following data were collected: age, sex, lesion to admission time (LTA); length of rehabilitation stay (LOS); total hospitalization duration (LTA + LOS). Injury variables (etiology, associated injuries, medical complications at admission and major surgical intervention) were recorded as dichotomous. The associated lesions were traumatic brain injury, severe facial injuries that affected the sense organs, major chest injury that required a chest tube or mechanical ventilation, abdominal injury, fractures of the pelvis or spine, and fracture of the upper or lower extremities.

Our criteria for discharging patients were when they reached sufficient independence with regard to everyday activities or when their functional status stabilized (no improvement from one month to another).

Neurological status included the American Spinal Injury Association (ASIA) standards [13]: motor scores (MS), neurological level, and ASIA Impairment Scale (AIS) grade. Patients were considered to have an incomplete lesion if they had motor or sensory function in the sacral segments (sacral sparing) [13]. Neurological recovery was defined, based on improvement in motor scores and AIS grade [2].

Functional status at admission and discharge was assessed by:

Barthel Index (BI) [14]: per standard protocols, scores from 0 to 100 were assigned at admission and discharge, with higher scores denoting greater levels of independence; Barthel Index subsets were also examined to identify areas of daily living that were more prone to be influenced by age (BI score was derived directly from the charts).

Rivermead Mobility Index [15], a 15-item mobility scale: the first 3 items of the scale evaluate a patient’s bed mobility and transfer, and the other 12 items assess walking; the scores range from 0 to 15 (full autonomy in bed motility, walking, and running). RMI score was derived directly from the charts.

Walking Index for Spinal Cord Injury II [16], a new scale that evaluates walking, based on the need for physical assistance, braces, and devices. The scale ranges from 0 (client unable to walk) to 20 (client walks without braces or devices and without physical assistance for at least 10 meters). Because WISCI scores were not in the original charts, they were retrospectively assessed from neurological clinical charts.

We categorized bladder control and autonomy in bowel management per previous studies [17]. Patients were divided into those who had normal bladder control and those with other emptying modalities. Normal bladder control was defined as follows:

patients can start and stop micturition at will

residual urine volume less than 100 ml, not requiring additional bladder emptying [18]

bladder pressure lower than 70–80 cm H2O (males) and 40–60 cm H2O (females) [19]

Per Shenot’s method [20], good bladder control was indicated also if patients needed medication to regulate bladder function. Thus, some patients were placed on low doses of anticholinergic agents to reduce bladder pressure and restore continence or on anti-alpha adrenergic drugs to help voiding.

We also categorized patients as having autonomy in bowel management or not. Bowel autonomy was defined as the ability to manage one’s bowels independently, adequate frequency of bowel movements (with or without drugs), and lack of fecal incontinence [17].

Finally, we recorded the incidence of complications during the rehabilitation stay and the patient’s destination at discharge.

## Statistical analysis

Descriptive data analysis: Descriptive values, expressed as mean + SD, were supplied for all continuous clinical data. Student’s *t* test for independent samples and chi square test were used to analyze differences between populations.

Poisson regression models were generated to assess the relative risks of patients with or without multiple injuries by characteristic (age, gender, lesion level, and AIS impairment). To evaluate outcomes, the relative risks (RRs) and 95% confidence intervals (CIs) were adjusted for the following confounders: lesion level and AIS grade. With regard to the level of the lesion, we divided patients into 3 groups: cervical, thoracic, and lumbar.

We also performed a separate analysis by student’s *T* test for independent samples to compare the outcomes between lesion levels (cervical, thoracic, and lumbar) and AIS grade groups.

Differences were considered significant if p < 0.05.

## Results

The study included 245 patients—202 males and 43 females; the mean age was 39.8 ± 17 years, and the mean lesion to admission time was 51.1 ± 58 days. A total of 136 patients presented with 263 multiple injuries (Table 1); thus, many patients were affected by more than 1 lesion.

**Table 1:** Frequency of associated lesions

	**Patients with cervical lesions N=32**	**Patients with thoracic lesions N=78**	**Patients with lumbar lesions N=26**	**Total**
Brain Injury	10	19	7	36
Chest and lung injury	19	60	13	92
Abdominal injury	8	14	9	31
Facial injury	5	8	4	17
Spine and pelvis injury	4	6	6	16
Upper extremity injury	10	25	13	48
Lower extremity injury	3	7	11	21
Total	59	141	63	263

The most frequent causes of trauma were motor vehicle accidents (51.4%), falls (27.7%), sports-related accidents (5.5%), and gunshot wounds and suicide attempts (4% each).

All but 25 patients (13 without multiple injuries and 12 with multiple injuries) underwent surgery for the spinal lesion.

Patients with multiple injuries had a significant prevalence of thoracic lesions (p<0.001) and complete lesions (p<0.05). They also presented more frequently with complications at admission (p<0.05) and had a longer LTA, rehabilitation LOS, and total hospitalization duration than monotraumatic subjects (Table 2).

**Table 2:** Clinical features of the two groups

	**Associated lesions**		**p**
	**No**	**Yes**	
**Age**	41±37.7	38±15.1	n.s.
**Lesion to admission time**	41.03±37.7	53.4±39.2	0,03
**Length of rehabilitation stay**	159.1±1	130.4±84.5	0,03
**Total hospital stay**	212.9±100.9	163.9±95.9	0.001
**Sex**			
Males	90	112	
Females	19	24	0,67
**Lesion level**			
Cervical	49	32	
Thoracic	28	78	
Lumbar-sacral	32	26	0.0001
**AIS grade at admission**			
A	43	74	
B	8	13	
C	28	31	
D	30	18	0.022
**Complications at admission**			
No	82	67	
Yes	27	69	0.0001

However, adjusting the outcomes for level and completeness of the lesion, patients with associated lesions had comparable functional and neurological status at admission and discharge, with similar BI, RMI, and WISCI and MS scores (Table 3) and percentages of independence in bladder and bowel function, frequency of complications, and discharge disposition (Table 4). At discharge, both groups had a mean BI score of approximately 60 points.

**Table 3:** Adjusted outcomes

	**Lesion**			**p-value**
	**No**	**Yes**		
	**mean (sd)**	**mean (sd)**	**RR**	
**BI admission**	23.3 (25.3)	16.7 (15.1)	0,989	n.s.
**BI discharge**	67.0 (31.7)	64.5 (26.1)	0,999	n.s.
**WISCI admission**	2.0 (5.1)	0.8 (3.5)	0,988	n.s.
**WISCI discharge**	8.8 (8.5)	5.3 (7.8)	0,987	n.s.
**RMI admission**	1.4 (2.9)	0.7 (1.2)	0,928	n.s.
**RMI discharge**	6.1 (4.6)	4.8 (1.0)	0,988	n.s.
**MS admission**	53.0 (22.6)	51.1 (17.3)	0,995	n.s.
**MS discharge**	63.0 (25.8)	57.7 (22.4)	0,994	n.s.

RR have been adjusted for lesion level and ASIA impairment at admission.

**Table 4:** Adjusted outcomes

	**Lesion**		**RR**	**p-value**
	**No**	**Yes**		
**Lenght of stay (Tertiles)**				
<100 days	45	35	1,00	
100-172	29	51	1,19	n.s.
>172	31	48	1,16	n.s.
**Total lenght of stay (Tertiles)**				n.s.
<120 days	29	17	1,00	n.s.
120-167	19	28	1,33	
167-210	15	29	1,38	
211-278	22	24	1,16	n.s.
>=279	12	33	1,62	
**Presence of pressare ulcers**				
	86	75	1,00	n.s.
Yes	23	61	1,35	
**Complications during stay**				
No	180	101	1,00	n.s.
Yes	56	43	1,40	
**Bladder emptying modalities**				
voiding modalities	29	38	1,00	n.s.
normal bladder control	80	98	1,03	
**Bowel voiding autonomy**				
No			1,00	n.s.
			0,99	
**Destination at discharge**				
	15	28	1,00	
Home	90	103	0,79	n.s.

RR have been adjusted for lesion level and ASIA impairment at admission.

With regard to length of stay, patients without multiple injuries tended to have a shorter length of stay (within 100 days) (Table 3). On assessing the total hospital stay duration (acute + rehabilitation), we noted that patients with multiple injuries had insignificantly longer stays.

In a separate analysis of each level of lesion and AIS grade, in some groups, patients with multiple injuries had a significant longer length of stay (Thoracic A and B, Lumbar A and B) or worse functional status at rehabilitation admission (Thoracic C and D, Lumbar A and B) than their monotraumatic counterparts (Tables 5, 6, 7).

**Table 5:** Comparison of patients with cervical lesions

	**Cervical A and B**			**Cervical C**			**Cervical D**		
	**Multiple injuries**		**p-value**	**Multiple injuries**		**p-value**	**Multiple injuries**		**p-value**
	**No**	**Yes**		**No**	**Yes**		**No**	**Yes**	
	**Mean (sd)**	**Mean (sd)**		**Mean (sd)**	**Mean (sd)**		**Mean (sd)**	**Mean (sd)**	
age	32.7 (18.4)	38 (15.8)	n.s.	53.8 (21.5)	49.8 (21.8)	n.s.	49.4 (19.6)	36.8 (15.6)	n.s.
LTA	36.2 (21.9)	60 (51.7)	n.s.	59.2 (52.5)	48.3 (21.4)	n.s.	39.1 (28.2)	49 (37.1)	n.s.
LOS	202.5 (106.2)	194.4 (120.8)	n.s.	148 (84.2)	172.9 (123.5)	n.s.	79.4 (51)	96 (37.2)	n.s.
LTA + LOS	225.6 (118.1)	253.9 (140.3)	n.s.	207.2 (97.3)	251.8 (141.7)	n.s.	118.3 (55.4)	146.50 56.959	n.s.
BI admission	2.2 (3.1)	1.94 (4.3)	n.s.	8.59 (12.9)	4.00 (5.5)	n.s.	41.3 (36.8)	32 (27.9)	n.s.
BI discharge	32.5 (31.4)	28.2 (29.1)	n.s.	50.2 (38.3)	52.6 (35.2)	n.s.	85.9 (16.7)	92.6 (6.3)	n.s.
BI improvement	32.3 (30.8)	27.1 (34.4)	n.s.	34.4 (36.2)	51.7 (45.4)	n.s.	45.6 (30.5)	62.1 (23.3)	n.s.
RMI admission	0.00	0.00	n.s.	0	0	n.s.	4.34 (4.1)	3.5 (4.8)	n.s.
RMI discharge	1.6 (2.7)	1.25 (2.6)	n.s.	5.4 (4.9)	5.5 (5.9)	n.s.	9.7 (2.8)	12.3 (2.4)	<0.05
RMI improvement	1.7 (2.8)	2 (3.8)	n.s.	5 (5.5)	7 (6.2)	n.s.	5.5 (3.4)	8.6 (4.1)	n.s.
WISCI admission	/	/		0.2 (0.9)	0	n.s.	7.1 (8.1)	6.6 (9.1)	n.s.
WISCI disharge	/	/		9 (7.6)	8.8 (9.9)	n.s.	18.5 (2.3)	19.2 (1.5)	n.s.
WISCI improvement	/	/		8.7 (7.5)	8.8 (9.9)	n.s.	11.4 (7.5)	12.6 (8.6)	n.s.

**Table 6:** Comparison of patients with thoracic lesions

	**Thoracic A and B**			**Thoracic C**			**Thoracic D**		
	**Multiple injuries**		**p-value**	**Multiple injuries**		**p-value**	**Multiple injuries**		**p-value**
	**No**	**Yes**		**No**	**Yes**		**No**	**Yes**	
	**Mean (sd)**	**Mean (sd)**		**Mean (sd)**	**Mean (sd)**		**Mean (sd)**	**Mean (sd)**	
age	43.84 (16.9)	38.07 (13.5)	n.s.	51 (14.8)	39.1 (16.8)	n.s.	39.5 (24.9)	38.4 (16.1)	n.s.
LTA	38.28 (33. 2)	46.88 (27.8)	n.s.	63.3 (53.8	63.1 (45.5)	n.s.	26.7 (11.6)	43 (18.7)	n.s.
LOS	142.58 (63.9)	183.39 (82.6)	n.s.			n.s.	46 (40.1)	77.8 (40.1)	n.s.
LTA + LOS	161.81 (82.2)	222.14 (89.4)	<0.05	162.3 (54.2)	219.4 (107.5)	n.s.	72.7 (37.1)	120.8 (46.5)	n.s.
BI admission	16.05 (7.3)	14.27 (5.7)	n.s.	38.7 (13.6)	15.9 (10.2)	<0.005	68.5 (27.8)	29 (15.3)	<0.05
BI discharge	56.62 (23.1)	59.48 (17.8)	n.s.	89.7 (2.5)	90.2 (11.9)	n.s.	95.5 (6.4)	89 (3.5)	n.s.
BI improvement	41.15 (22.4)	38.89 (19.1)	n.s.	45 (3.8)	67.5 (17.7)	n.s.	55 (7.1)	72.5 (3.5)	n.s.
RMI admission	0.25 (0.9)	0.16 (0.5)	n.s.	1.7 (1.5)	0.3 (0.8)	<0.05	7 (7)	1.6 (1.1)	n.s.
RMI discharge	2.85 (1.7)	2.73 (1.3)	n.s.	7.3 (0.6)	7.2 (3.4)	n.s.	13.7 (1.5)	9.8 (4.4)	n.s.
RMI improvement	2.77 (1.9)	2.46 (1.5)	n.s.	8 (1.6)	7.4 (4.1)	n.s.	10 (2.8)	9.5 (4.4	n.s.
WISCI admission	**/**	**/**		0	0	n.s.	9.2 (8.9)	2.6 (5.8)	n.s.
WISCI disharge	**/**	**/**		16 (3)	10.8 (6.5)	n.s.	19.7 (0.5)	18.4 (2.2)	n.s.
WISCI improvement	**/**	**/**		19 (1.3)	12.2 (6.9)	n.s.	10.5 (9.1)	15.8 (7.4)	n.s.

**Table 7:** Comparison of patients with lumbar lesions

	**Lumbar A and B**			**Lumbar C**			**Lumbar D**		
	**Multiple injuries**		**p-value**	**Multiple injuries**		**p-value**	**Multiple injuries**		**p-value**
	**No**	**Yes**		**No**	**Yes**		**No**	**Yes**	
	**Mean (sd)**	**Mean (sd)**		**Mean (sd)**	**Mean (sd)**		**Mean (sd)**	**Mean (sd)**	
	37.5 (16.4)	37.2 (15.6)	n.s.	31.3 (12.4)	33 (14.9)	n.s.	41.5 (18.3)	25 (2.6)	n.s.
LTA	21 (7.6)	69.8 (60.6)	<0.05	56.3 (72.5)	53.6 (48.3)	n.s.	36.5 (34)	57.3 (17.2)	n.s.
LOS	144.7 (52.3)	155 (78.9)	n.s.	123.6 (115.1)	89.8 (50.4)	n.s.	63.2 (19.8)	80.7 (30.1)	n.s.
LTA + LOS	160.8 (53.4)	224.8 (103.9)	n.s.	179.9 (147.5)	148.7 (65.7)	n.s.	91.4 (49.9)	138 (45.1)	n.s.
BI admission	19.5 (8.6)	23.8 (15.8)	n.s.	27.7 (18.3)	24.6 (20.3)	n.s.	49.4 (25.4)	43.3 (15.3)	n.s.
BI discharge	79.5 (8.1)	70 (20.2)	n.s.	71.3 (20.8)	70.7 (18.2)	n.s.	90.4 (9.8)	89.7 (8.4)	n.s.
BI improvement	62.4 (6.2)	46.9 (19.2)	<0.05	61 (26.3)	49.3 (33.2)	n.s.	45 (27.3)	43.3 (15.3)	n.s.
RMI admission	0.3 (0.9)	1.1 (1.5)	n.s.	1.3 (2.1)	0.7 (1.5)	n.s.	4.5 (3.1)	2.3 (0.6)	n.s.
RMI discharge	7 (3.2)	5.3 (2.5)	n.s.	7.4 (3.2)	6.1 (3.4)	n.s.	11.2 (3.7)	8 (2.7)	n.s
RMI improvement	6.7 (3.2)	4.2 (1.8)	<0.05	6.7 (4.2)	5.4 (3.5)	n.s.	6.7 (4.1)	5.7 (2.8)	n.s.
WISCI admission	0	0	n.s.	0	0	n.s.	7 (7.2)	9.7 (8.5)	n.s.
WISCI disharge	8 (6.1)	3.9 (5.7)	n.s.	10.1 (8.1)	9 (7.3)	n.s.	16.1 (5.8)	18.7 (2.3)	n.s.
WISCI improvement	8 (6.1)	3.9 (5.7)	n.s.	10.1 (8.1)	9 (7.3)	n.s.	9.1 (5.9)	9 (6.2)	n.s.

## Discussion

The impact of associated lesions on the functional and neurological status of SCI patients remains controversial. Hebert [7] examined the impact of multiple injuries in spinal fractures and spinal cord injuries on the use of resources and reported a negative impact with higher costs and longer hospital length of stay. Recently articles compared the outcomes of SCI patients with traumatic and nontraumatic lesions [21], observing that patients with traumatic SCIs had worse functional status at admission than their nontraumatic counterparts and needed a longer length of stay to reach comparable levels of independence. The authors attributed these differences to non-neurological, trauma-related factors, such as major surgery-related sequelae, the need to wear an orthotic, and multiple injuries—some of these factors have an influence on the status of patients [22]. In the same study, traumatic patients had a higher frequency of multiple injuries and major surgery.

However, 2 recent articles by Putz [10,11] demonstrated in a small cohort of patients with complete lesions at the thoracic level that subjects with multiple injuries have the same neurological (percentage AIS improvement) and functional outcomes as those without multiple injuries, although the former experienced a slower recovery of daily life independence. Conversely, Macciocchi [12] examined the effects of co-occurring brain injury and found that SCI patients with paraplegia and severe traumatic brain injury had lower FIM Motor Scale scores at admission and discharge and a longer rehabilitation LOS than patients without brain injury or mild brain injury.

Based on these findings, we felt compelled to examine the impact of multiple injuries in a large cohort of patients, including those with cervical and lumbar lesions and incomplete lesions.

The demographics of our series are similar to those of the European traumatic population, for whom road accidents and falls being are the most frequent [23]. Contrary to our initial hypothesis, we failed to observe a negative effect of multiple injuries on the outcomes of SCI patients, thus confirming the findings of Putz [10,11]. Based on the initial evaluation of the 2 cohorts (Table 1), patients with multiple injuries appeared to have worse functional status at admission and discharge and more complications at admission than monotraumatic subjects.

However, patients with multiple injuries presented with significant differences as with a higher percentage of complete lesions and thoracic lesions. As level and completeness of the lesion are the main factors influencing the prognosis of SCI, we used a statistical method to correct for their influence. On comparing the 2 cohorts by Poisson regression model, the differences in functional outcome at admission and discharge disappeared.

With regard to neurological outcomes, both populations achieved comparable ASIA motor scores. These data are consistent with those of Putz, who showed that multiple injuries have no effect on the likelihood of neurological recovery and that neurological recovery is primarily related to the completeness of the lesion.

For functional status, at admission and discharge, BI, RMI, WISCI, and MS scores were comparable between the 2 populations, as was bowel and bladder control. However, in a separate analysis of each level of the lesion and AIS grade, in certain groups (Thoracic C and D, Lumbar A and B), patients with multiple injuries had worse functional status at admission to rehabilitation than their monotraumatic counterparts. With regard to functional recovery, our study has recapitulate the finding that the prognosis of SCI patients depends primarily on AIS grade at admission and the lesion level, whereas the presence of multiple injuries does not influence functional outcomes in SCI patients. The effects of lesion level and completeness are intuitive and have been reported several times [4,24].

With regard to discharge destination, the 2 populations had a similar percentage of returning home, although patients with multiple injuries tended to be discharged more frequently to other rehabilitation facilities or hospitals, due to the good functional status that was reached by the patients. At discharge, multiply injured and monotraumatic patients had a mean BI score of approximately 60 points, a pivotal score at which patients transit from dependence to assisted independence and that can be considered a cutoff for discharge home [25].

With regard to length of stay, our cohort and that of Putz were not comparable as to baseline examination: her patients were evaluated within 2 weeks of the injury, whereas our patients were seen at a mean of 50 days after the injury. To avoid this bias, we calculated the total duration of hospitalizations to generate data that approximated those of Putz more closely. In the entire group of patients we failed to observe any statistical differences between cohorts, although patients with multiple injuries tended to have longer hospital stays. In groups with certain lesion levels and AIS grades (Thoracic A and B, Lumbar A and B), the difference was significant.

This study has several shortcomings that deserve further analysis, primarily due to its retrospective nature. The stratification of lesion levels in cervical, thoracic, and lumbar lesions could be misleading, because the outcome of high tetraplegia and high paraplegia differ from those of low tetraplegia and low paraplegia. A more detailed stratification is recommended, but the number of subjects in our study did not permit further discrimination.

Further, we categorized the presence of multiple injuries dichotomously (yes or no), without adopting a scoring system as Putz did [10,11] and without subdividing the lesions. It is possible that certain specific lesions were associated with a worse outcome, as reported. In particular, concomitant brain injuries would need a separate analysis [12].

## Conclusions

Multiply injured patients are affected by more severe neurological lesions. However, when the effects of lesion features (level and completeness) are eliminated with an appropriate statistical method, the SCI patients with and without multiple injuries have comparable outcomes. These data confirm that the principal determinants of outcomes in SCI patients are the level and completeness of the lesion.

## Competing interests

The authors declare no conflict of interest; supported in part by grant RC12G of the Italian Ministry of Health and grant P133 of the International Foundation for Research in Paraplegie to GS.

## Authors’ contribution

GS designed the study and contributed to data collection and manuscript writing. SF performed the statistical analysis. LL and FT contributed to data collection and manuscipt writing. MM participated in the coorination of the study and helped drafting the manuscript. All authors read and approved the final manuscript.

## Aknowledgments

The professional editing of Blue Pencil is gratefully acknowledged.
